# High pathogenicity avian influenza: targeted active surveillance of wild birds to enable early detection of emerging disease threats

**DOI:** 10.1017/S0950268822001856

**Published:** 2022-12-11

**Authors:** Daniel Wade, Adham Ashton-Butt, Graham Scott, Scott M. Reid, Vivien Coward, Rowena D. E. Hansen, Ashley C. Banyard, Alastair I. Ward

**Affiliations:** 1 Department of Biological and Marine Sciences, University of Hull, Cottingham Road, Hull, HU6 7RX, UK; 2 British Trust for Ornithology, The Nunnery, Thetford, IP24 2PU, UK; 3 Virology Department, Animal and Plant Health Agency, Weybridge, New Haw, Surrey, KT15 3NB, UK; 4 School of Biology, University of Leeds, Woodhouse Lane, Leeds, LS2 9JT, UK

**Keywords:** Bird flu, disease surveillance, epidemiology, migration ecology, ornithology, zoonotic

## Abstract

Avian influenza (AI) is an important disease that has significant implications for animal and human health. High pathogenicity AI (HPAI) has emerged in consecutive seasons within the UK to cause the largest outbreaks recorded. Statutory measures to control outbreaks of AI virus (AIV) at poultry farms involve disposal of all birds on infected premises. Understanding of the timing of incursions into the UK could facilitate decisions on improved responses. During the autumnal migration and wintering period (autumn 2019– spring 2020), three active sampling approaches were trialled for wild bird species considered likely to be involved in captive AI outbreaks with retrospective laboratory testing undertaken to define the presence of AIV.

Faecal sampling of birds (*n* = 594) caught during routine and responsive mist net sampling failed to detect AIV. Cloacal sampling of hunter-harvested waterfowl (*n* = 146) detected seven positive samples from three species with the earliest detection on the 17 October 2020. Statutory sampling first detected AIV in wild and captive birds on 3 November 2020. We conclude that hunter sourced sampling of waterfowl presents an opportunity to detect AI within the UK in advance of outbreaks on poultry farms and allow for early intervention measures to protect the national poultry flock.

## Introduction

Avian influenza (AI) is caused by a zoonotic viral pathogen (Influenza A virus, AIV) hosted predominantly by wild birds but with the ability to jump to other taxonomic groups [1], including humans. AIV is divided, based on infection in poultry into high pathogenicity (HPAIV) and low pathogenicity (LPAIV) with outcomes resulting from infection with the former being associated with high mortality whilst LPAIV infection is invariably asymptomatic in poultry [2]. Further, within the UK, the detection of H5 and H7 virus subtypes is legally notifiable and impacts both on national AIV status and international trade. Globally, the annual number of HPAIV cases on poultry farms and among wild birds caused by H5Nx viruses has increased in recent years, with substantial increases observed during 2020/21 and 2021/22 [3]. Between 2017 and the end of 2019, only 40 AIV – positive wild birds were detected in Great Britain (GB). In each of the following autumnal migration and wintering periods, the incidence of wild bird detections increased substantially (317 in 2020/2021 and as of the 21 July 2022, 1413 in 2021/2022 where multiple summer peaks were observed following the end of winter) with the trend similarly mirrored in poultry farm AI outbreaks [4]. The cost to the industry of statutory measures to control AI on UK poultry farms between 2016 and 2017 was estimated to exceed £100 million, with additional cost to the government for monitoring and outbreak control [5].

UK poultry farmers are legally obliged to report suspicion of AIV infection within their flocks, which is followed by testing and implementation of control measures if notifiable AIV is confirmed [6]. Detection of AIV in wild birds is passive, relying on submission of found-dead bird carcasses for testing by the UK statutory agency [7]. Under these approaches, detection of AIV in UK poultry and wild birds has been approximately concomitant. However, it may be advantageous to detect re-emergence of infection in the country among wild birds before the first outbreaks among poultry. In this way, enhanced biosecurity measures, and informed decisions on housing free-range poultry, could be implemented early in order to attempt to reduce the risk and frequency of AI outbreaks.

The primary wild host for both LP and HPAVI is thought to be the *Anatidae*; ducks, geese and swans [8] with annual re-emergence of AI on European poultry farms following soon after their seasonal immigration [9]. However, many species of passerine have been found to carry AI and have also been proposed as potential candidates for direct exposure to poultry [10]. Substantial variation in the prevalence of infection among passerines and other non-*Anatidae* bird families has been found between studies [11–18]. The mechanism by which AIV transmits from migrational *Anatidae* into poultry is unclear but transfer via intermediate bridge species, which may include passerines, has been proposed [19]. Confirmation of the same strains of AIV infecting wild passerines and domestic poultry concomitantly in space and time could help further elucidate the mechanisms of transfer of AIV from wild to domestic birds.

Hundreds of thousands of individual passerines (and other birds) are ringed (fitted with uniquely coded rings upon the tarsus or tibia) across the UK as part of voluntary national monitoring of bird populations overseen by the British Trust for Ornithology [20]. Furthermore, more than 140 wildfowling clubs with over 9000 members, lawfully shoot ducks and geese every year for recreational purposes [21]. These two activities offer potential for AI surveillance in wild bird populations due to their temporal and spatial coverage and the potential sample sizes being very large. This study sought to evaluate whether active sampling of wild birds could result in the detection of AIV in advance of outbreaks on poultry farms. The study chose to sample birds that were temporarily (bird ringing) or permanently (shooting) removed from the wild during lawful routine activities to establish whether these activities might offer opportunities for cost-effective AI surveillance. Also sampled were birds caught and ringed at established ringing sites at locations local to a poultry farm that had recently experienced an AI outbreak to identify whether the same strains of AIV were detectable in wild birds likely to visit those farms, and to evaluate the potential for highly targeted surveillance of AIV in wild bird populations.

## Methods

Three approaches were tested to evaluate their ability to detect AIV in wild birds between Autumn 2019 and Spring 2021: (1) sampling of hunter-harvested waterfowl, (2) volunteer sampling of migrating birds and (3) responsive sampling of birds caught close to a poultry farm AI outbreak.

Cloacal swab samples were collected *post mortem* from hunter-harvested waterfowl at a private site on the northern side of the outer Humber Estuary in northeast England (Lat/Long, 53.653476, 0.073939). The site was chosen because of its position on the east coast of the UK, where many migratory birds enter the country during their autumn migration [22].

**Fig. 1.**
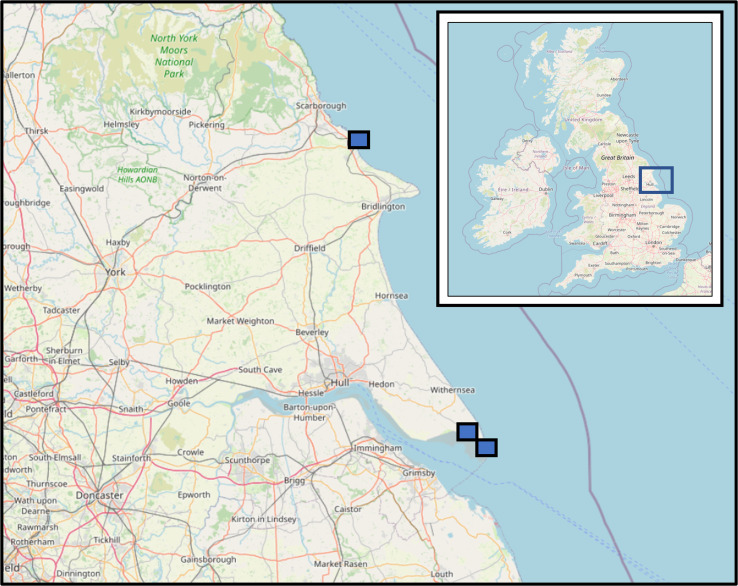
Locations of migratory bird sampling sites at Filey Brigg (a), Welwick saltmarsh (b) and on the Spurn peninsula (c) within the UK [23].

Faeces passed by migratory birds upon capture were collected in the same region at three sites with ongoing bird banding/ringing projects during autumn 2019 and 2020 (Fig. 1).

Faecal samples were collected from passerines caught in mist nets from one hour before dawn until catches became minimal during daylight hours. Mist nets were used under licence from the British Trust for Ornithology (BTO) and birds were extracted and placed singly into a cloth bag from where faecal samples were collected into 1.5 ml screw cap microcentrifuge tubes. Tubes were labelled with a unique sample code, the date, species of origin and the number of any ring present or fitted during the bird's capture.

Each cloth bag was only used once per sampling session to avoid cross contamination of samples, and bags were soaked in a weak bleach solution [24] and washed at a high temperature before subsequent use to deactivate AIV and prevent its amplification by RRT-PCR.

Wading birds were caught monthly in mist nets over autumn and winter 2019/2020 and 2020/2021 during nights with a waning or new moon to limit the ability of waders to see the nets. Mixed species vocalisation play backs were used to attract waders to the catching areas. Upon capture, waders were placed into single species holding crates, which were lined with plain paper. Once all birds were processed (biometrics taken and ringed), individual faecal samples were collected from the boxes and placed into 1.5 ml microcentrifuge tubes labelled by batch number (for which the ring numbers of birds in each batch were recorded), species and date. Lining paper was replaced between each batch to avoid cross contamination.

A single duck trap was placed on Kilnsea Wetlands (Spurn peninsula) and baited with grain for ducks during the winter 2020/2021. Further, dead birds found in the wider Spurn area were collected, and sampled for AIV via a cloacal swab.

**Fig. 2.**
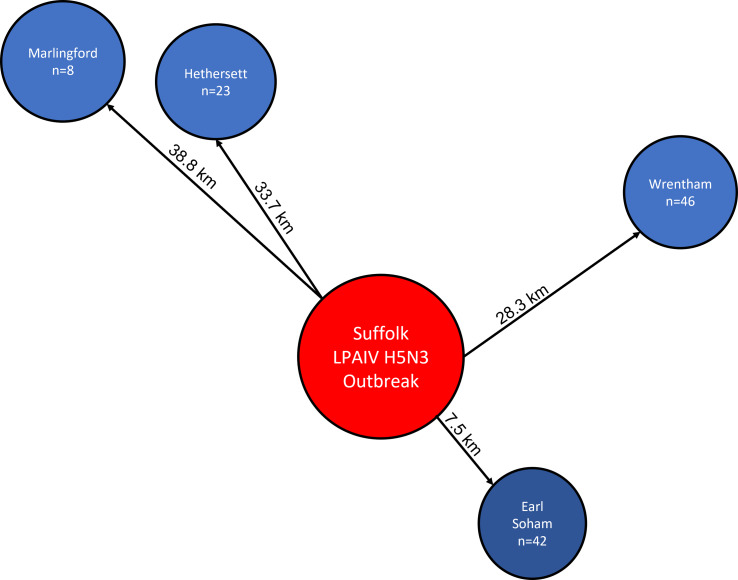
Faecal sampling locations in response to December 2019 LPAI H5N3 captive outbreak near Athelington, Mid-Suffolk. No sampling took place at the outbreak site due to 1 km exclusion zone.

On 7th December 2019, low pathogenicity H5N3 was confirmed on a commercial poultry farm in mid-Suffolk as part of the UK notifiable disease investigation process. In response to this outbreak, passerines were captured at bird ringing sites (see Fig. 2) using mist nets, and faecal samples were collected, as described above.

The Animal and Plant Health Agency (APHA) publishes data on AIV detections in wild birds [25] in addition to poultry outbreak reports [4]. From these, data on detection time and species were extracted to compare with data collected during active sampling.

All swabs and faecal samples were stored dry in 35 ml centrifuge tubes and placed into a chest freezer at −20 °C within 24 h and then stored within a −80 °C freezer within 4 days of sample collection. Samples were transported on dry ice within 24 h of removal from −80 °C storage for virological investigation at the APHA in Weybridge, Surrey. Samples were retrospectively tested with the Nagy matrix (M)-gene detection real-time reverse transcription polymerase chain reaction (RRT-PCR) for generic detection of AIV RNA [26]. Positive samples were then tested by H5-specific RRT-PCR [27]. Samples testing positive by H5-specific RRT-PCR were further tested by a high pathogenicity H5 detection RRT-PCR [28] to confirm the presence of HPAIV H5 in these samples.

To compare the timing of AIV incursion detected by each of the sampling approaches with bird migration trends, data on relative abundance (% of sites in the UK where a species was present in any given week) were downloaded from eBird's Basic Dataset (EBD), a downloadable citizen science repository for bird sightings [29].

## Results

### Active sampling of hunter-harvested wildfowl

Between 18 October 2020 and 13 January 2021, cloacal swabs were collected from 146 shot birds from 7 different species of waterfowl. A total of 7 shot birds tested positive for AIV (Table 1).

**Table 1.** Results from cloacal swab sampling for detection of all strains of AIV in hunter-harvested waterfowl on the Humber Estuary, UK

Species of waterfowl	Number of samples collected	Number of Positive Samples (% of samples)
Mallard (*Anas platyrhynchos*)	12	1 (8.3%)
Northern Shoveler (*Spatula clypeata*)	2	0
Eurasian Teal (*Anas crecca crecca*)	101	4 (4.0%)
Eurasian Wigeon (*Mareca penelope*)	23	2 (8.7%)
Greylag Goose (*Anser anser*)	4	0
Canada Goose (*Branta canadensis*)	1	0
Pink-footed Goose (*Anser brachyrhynchos*)	3	0
*Total*	*146*	*7 (4.8%)*

Low pathogenicity H5 was detected in Teal (1) and highly pathogenicity H5 was detected in Eurasian Teal (1) and Eurasian Wigeon (1) (Table 2).

**Table 2.** H5 strain and highly pathogenic H5 identification results from retrospective PCR typing from cloacal swab sampling in hunter-harvested waterfowl on the Humber Estuary, UK

Species	Date of sample collection	H5 HA2 result [26]	HP H5 [27]
Eurasian Teal	18^th^ October 2020	−	−
Eurasian Teal	19^th^ October 2020	+	−
Eurasian Wigeon	31^st^ October 2020	+	+
Mallard	11^th^ November 2020	−	−
Eurasian Teal	11^th^ November 2020	+	+
Eurasian Wigeon	28^th^ November 2020	−	−
Eurasian Teal	28^th^ November 2020	−	−

‘+’ = positive, ‘−‘ = negative.

**Fig. 3.**
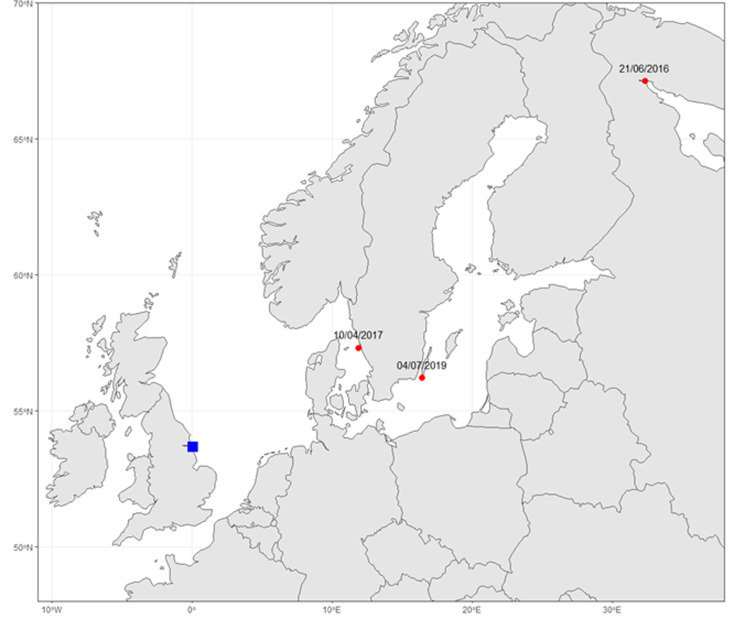
Locations of ringing location of already ringed Eurasian Teal shot on the Humber Estuary during winter 2020/2021.

Among teal, one shot on 19 October 2020 had been ringed on the 10 April 2017 at Nidingen, Halland, Sweden, one shot on 28 November 2020 had been ringed at Ottenby, Öland, Sweden on 4 August 2019, and another shot on 28th November 2020 had been ringed in Murmansk Oblast, Russia on 21 July 2016 (see Fig. 3).

### Active faecal sampling of immigrant birds

A total of 475 faecal samples from 34 species were collected during autumn migration between October 2019 and November 2020. The majority (*n* = 382) were collected at Spurn peninsula and 66 were collected from Filey Brigg (Table 3).

**Table 3.** List of faecal samples collected by location, year and species during active migration sampling for AI

Species	Filey	Spurn		Total
	2019	2019	2020	
Blackbird (*Turdus merula*)	3	20	1	24
Blackcap (*Sylvia atricapilla*)	3	6	16	22
Blue Tit (*Cyanistes caeruleus*)	13	0	1	14
Brambling (*Fringilla montifringilla*)	1	0	0	1
Bullfinch (*Pyrhulla pyrhulla*)	1	0	0	1
Chaffinch (*Fringilla coelebs*)	2	3	3	8
Chiffchaff (*Phylloscopus collybita*)	0	0	4	4
Coal Tit (*Periparus ater*)	2	0	0	2
Common Whitethroat (*Curruca communis*)	0	0	1	1
Dunnock (*Prunella modularis*)	3	0	9	12
Goldcrest (*Regulus regulus*)	2	2	8	12
Goldfinch (*Carduelis carduelis*)	0	2	4	6
Great Spotted Woodpecker (*Dendrocopos major*)	0	1	0	1
Great Tit (*Parus major*)	2	2	0	4
Greenfinch (*Chloris chloris*)	3	0	0	3
House Sparrow (*Passer domesticus*)	0	4	1	5
Lesser Redpoll (*Acanthis flammea cabaret*)	0	0	55	55
Linnet (*Linaria cannabina*)	0	0	2	2
Long-tailed Tit (*Aegithalos caudatus*)	1	0	0	1
Meadow Pipit (*Anthus pratensis*)	0	0	77	77
Red-flanked Bluetail (*Tarsiger cyanurus*)	1	0	0	1
Redstart (*Phoenicurus phoenicurus*)	0	0	2	2
Redwing (*Turdus iliacus*)	3	11	2	16
Reed Bunting (*Emberiza schoeniculus*)	1	1	12	14
Reed Warbler (*Acrocephalus scirpaceus*)	0	1	0	1
Robin (*Erithacus rubecula*)	7	4	23	34
Sedge Warbler (*Acrocephalus schoenibaenus*)	0	0	1	1
Siskin (*Spinus spinus*)	1	4	1	6
Song Thrush (*Turdus philomelos*)	1	5	4	10
Starling (*Sturnus vulgaris*)	0	0	1	1
Tree Sparrow (*Passer montanus*)	6	54	30	90
Willow Warbler (*Phylloscopus trochilus*)	0	1	1	2
Wren (*Troglodytes troglodytes*)	5	1	1	7
Yellow-browed Warbler (*Phylloscopus inornatus*)	0	1	2	3
Yellowhammer (*Emberiza citrinella*)	1	0	0	1
Total	66	146	262	474

Faecal samples (*n* = 12) were also collected from 3 species of wader: bar-tailed godwit (*Limosa lapponica*, *n* = 1), redshank (*Tringa tetanus, n* = 10) and knot (*Calidris canutus*, *n* = 11). The duck trap caught a small sample of waterfowl: mallard (*Anas platyrhynchos*, *n* = 4) and shoveler (*Spatula clypeata*, *n* = 1). Finally, four birds were sampled when discovered dead or weakened at Spurn: one each of cormorant (*Phalacrocorax major*), whooper swan (*Cygnus cygnus*), mute swan (*Cygnus olor*) and common scoter (*Melanitta nigra*). None of the active faecal migration samples tested positive for AIV.

### Responsive faecal sampling

Faecal samples (*n* = 119) were collected from 16 species at 4 sites between 7.5 and 38.8 km of the outbreak site (see Fig. 2) as an AI infected poultry farm (Table 4), but none tested positive for AIV.

**Table 4.** The number of faecal samples collected per species during outbreak responsive sampling for AI in Norfolk and Suffolk

Species	Number of Samples
Blue Tit (*Cyanistes caerulus)*	59
Great Tit (*Parus major*)	7
Dunnock (*Prunella modularis*)	11
Blackbird (*Turdus merula*)	4
Bullfinch (*Pyrrhula pyrrhula*)	1
Chaffinch (*Fringilla coelebs*)	3
Coal Tit (*Periparus ater*)	2
Goldcrest (*Regulus regulus*)	1
Goldfinch (*Carduelis carduelis*)	1
House Sparrow (*Passer domesticus*)	2
Long-tailed Tit (*Aegithalos caudatus*)	3
Red-legged Partridge (*Alectoris rufa*)	1
Reed Bunting (*Emberiza schoeniclus*)	3
Robin (*Erithacus rubecula*)	9
Wren (*Troglodytes troglodytes*)	2
Yellowhammer (*Emberiza citrinella*)	7
Total	119

### Passive sampling

During autumn/winter of 2020/2021, HPAI H5N8 AIV was first confirmed in the UK by passive surveillance of wild birds on the 9 November 2020. The birds (a greylag *Anser anser* and Canada goose *Branta canadensis*) had been found dead on the 3 November 2020. Over the outbreak season (November 2020–April 2021), 311 of 1345 different wild bird carcasses from 22 species tested positive for AIV from locations across the UK [30].

**Fig. 4.**
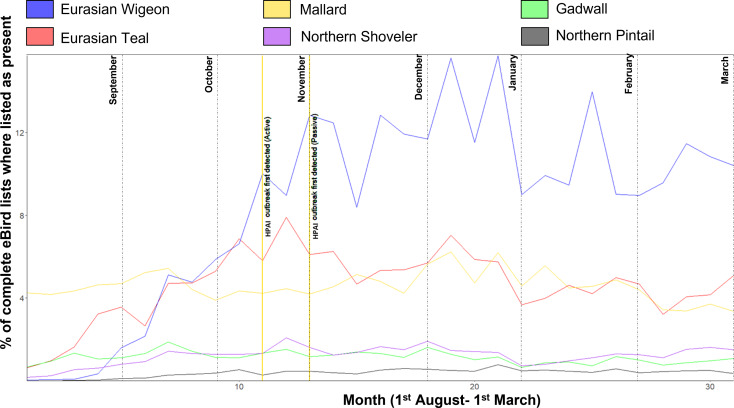
Relative abundance (% of complete ebird species presence checklists where present per week) of migratory dabbling ducks in the UK from the 1 August 2020 to 1 March 2021 constructed from Ebird abundance data [29]. First detection dates for both passive and active methods are shown and labelled in vertical yellow lines. Month lines signify the week that included the first day of the month.

Waterfowl migration into the UK during autumn 2020 (Fig. 4) varied by species but increases in numbers of teal began in mid-August, with the highest peak witnessed during the last week of October. Eurasian Wigeon were most abundant with the first influxes detected at the end of August and peaking during December [29].

## Discussion

During the autumn/winter of 2020/21 LPAI was retrospectively confirmed in a Eurasian teal that had been shot and sampled on 19 October, a full 11 days before LPAI was first detected as part of a statutory notifiable avian disease investigation following a non-negative result from active serological surveillance within poultry. Further, HPAI was retrospectively detected in a hunter-harvested Eurasian wigeon that was shot on 31 October, three days before HPAI was first detected on a poultry farm and three days before HPAI was detected in wild birds as part of the UK AIV passive surveillance scheme (which was confirmed 6 days later). Despite a small sample size (*n* = 152), 7 ducks tested positive for AIV in 2020 in comparison with no positive results obtained from 474 samples collected from passerines during 2019 and 2020. Moreover, none of 119 samples collected within the locality of an AI infected poultry farm tested positive for AIV. These results further support priority surveillance for AIV in *Anseriformes*. The percentage of hunter-harvested samples testing positive for HPAIV was surprisingly consistent with the results of a study involving 4729 hunter-harvested birds in the USA during 2014-15; 1.3%, with Eurasian teal and Eurasian wigeon prevalent within the sample [31].

Use of a walk-in duck trap was proposed to be used to contrast with the other active sampling methodologies, but this element was constrained by delays and sampling only began into the wintering period producing a small number of samples. Other sites within the UK and abroad have used this method for bird ringing of waterfowl (producing a larger sample size [8]) so, whilst not demonstrated during the current study, live duck traps could offer an alternative active sampling method.

A similar study was conducted in wild waterfowl at two sites in northern Italy between November 2020 and January 2021 where 823 hunted and 521 live captured ducks were sampled (cloacal and orophangyeal). Results demonstrated higher AIV prevalence than was detected on the Humber, with 6.7% of sample positive for AIV in hunter-harvested birds and 9.7% in samples from birds that were captured and released, compared with 4.8% on the Humber. Whilst AIV detection was most frequent in northern Italy during November and January, different peaks were evident between the two sets of samples. Week 49 and 50 (of the year) showed the largest number of positive samples for live captured birds and week 47 was highest for hunter harvested birds (though no birds were sampled by this method in week 49). Whilst no live captured birds tested positive after the 1^st^ week of January, 5 hunter harvested birds tested positive in the last week of the study (week 4 of the year) indicating that a longer study period may reveal more about changing AIV prevalence in different locations [32].

The active sampling methods in the current study were highly spatially focussed in comparison with the UK's current passive surveillance of found-dead birds, but these approaches offered the additional advantage of sampling clinically healthy individuals as well as those that had yet to develop symptoms of disease. Asymptomatic but transmissible infections of AIV have been detected in waterfowl and other avian species during challenge experiments and it is plausible that the same findings may be seen in wild birds [33]. The importance of infected asymptomatic waterfowl in AIV epidemiology has yet to be fully evaluated [34].

Passive monitoring did detect infection among wild birds across the UK, but no spatio-temporal pattern was discernible. Most migratory bird species enter the UK from breeding grounds to the east (Scandinavia, Central and Eastern Europe, Arctic Russia), but the first passive detection within British wild birds was recorded in Gloucestershire, in the southwest of the UK. If *Anatidae* species are predominantly responsible for the seasonal re-emergence of AIV [35], then it seems likely that the earliest detection of infection is most likely to occur at east coast locations that attract large numbers of immigrant waterfowl, such as the Humber Estuary. Early detection of AIV offers opportunities to better understand the dynamics of the disease [31] and to advise enhanced biosecurity practices among poultry farmers. However, detection of AIV from a larger, more geographically-dispersed sample size over a longer study period would be required to afford greater confidence in the ability of surveillance of hunter harvested *Anatidae* to reliably indicate the seasonal re-emergence of AIV within the UK. Furthermore, similar methods used along the migration pathways of these species would further aid in tracking international AIV dynamics.

Detection of the same strain of AIV among wild birds that occupy poultry farms could help identify those species that pose the greatest risks to poultry [15]. However, the responsive sampling of birds on land close to a farm experiencing an outbreak of AIV yielded no samples positive for AIV, probably due to small sample size and too great a distance from the farm. The minimum distance was a statutory limitation and could not have been overcome. At present, any sampling (wild bird or otherwise) for AIV within 5 km of an outbreak in poultry can only be performed by trained APHA personnel.

Previous studies investigating AIV prevalence in passerines have mostly detected low levels or no AIV within their samples, though the numbers of studies focussing on wild passerines is heavily outweighed by those focussing on *Anatidae*. However, this is not universal, with Gronesova *et al*. [16] detecting 16% prevalence in both oropharyngeal and cloacal swabs from summering birds at a reedbed site in Slovakia. Han *et al*., [18] reported no AIV positive samples from rectum eluate from 1300 tree sparrows (*Passer montanus*) but 94/800 seropositive samples from the same species indicating that although 94 individuals had been immunologically challenged by AIV, none were actively excreting at the time of sampling. An extensive US study [15] involving the collection of cloacal samples at ringing stations found that AIV prevalence was higher in passerines than in 8 other sampled orders (*n* = 13 046). Whilst there will likely be differences in the epidemiological network between the new and old world (different species and families), a UK or flyway-wide study of similar magnitude may be required to clarify the potential roles of passerines in AIV epidemiology in Eurasia.

Active sampling of hunter-harvested waterfowl is limited to certain species that can be lawfully harvested (see below) and by the UK open season which covers the period from the September 1^st^ to January 31^st^ under all devolved administrations except the Isle of Man. This extends to February 20^th^ in England, Wales and Scotland when hunting below the high-water mark. Legal quarry also limits what can be sampled. Gadwall (*Mareca strepera*), common goldeneye (*Bucephala clangula*), mallard, northern pintail (*Anas acuta*), common pochard (*Aythya ferina*), northern shoveler, Eurasian teal, tufted duck (*Aythya fuligula*) and Eurasian wigeon are legal quarry for ducks and Canada goose, greylag goose, pink-footed goose (*Anser brachyrhynchus*) and European white-fronted goose (*Anser albifrons*) can all be lawfully shot, but other duck and goose species and all swans cannot [36]. These are the most abundant land-based species within the family of *Anatidae* in the UK with the possible exception of barnacle geese (*Branta leucopsis*) [37]. The restrictions of the hunting season, whilst clearly important from a wildlife conservation perspective, limit the ability to utilise this method as a year-round approach to AIV surveillance, and thus is most relevant to detection of autumnal influxes and overwinter fluctuations of AIV in legally huntable waterfowl. Whilst this study has assessed sampling methods for AIV, other avian zoonotic diseases of anthropocentric concern, such as Newcastle disease, could be monitored through a similar scheme.

UK autumn migration in dabbling ducks rose in mid-late August varying by species, with most wintering birds present by mid to late November (peaks for Eurasian teal in October, Eurasian wigeon and mallard in December [29]), but the hunter-harvested active sampling protocol was only implemented from mid-October. Consequently, AI may have been present on the Humber estuary in wild birds before the actual initial detection date. Future research to identify the earliest date of incursion of AI into the UK via wild birds should start sampling Anatidae from 1st September, obtain much larger samples size from a wider distribution of locations. A more precise assessment of new strains of AIV present in wild birds could inform the timing of enhanced and targeted biosecurity practices on poultry farms and captive flocks and hence has potential to enhance preparations for AIV incursions and subsequently reduce the impact of AI during the peak season. Poultry holdings lose their free-range status during winter periods during enforced biosecurity lockdown of free-ranging flocks, which might affect consumers' purchasing decisions.

Bevin *et al*. [31] and Gobbo *et al*. [32], have also argued that the hunter network offers a potentially cost-effective approach to AI monitoring. This study has shown with a single sampling site that it was possible to detect AIV in the UK via an active sampling approach before a nationwide passive approach did. Utilisation and expansion of a hunter harvested AI surveillance network may provide the UK with an alternative to its current passive surveillance and could allow for important increases in time between AIV detection in the wild and captive environments. This would allow for increasingly informed decisions on suitable AI mitigation and further understanding of AI dynamics during wild outbreaks.

## Data Availability

Data supporting this study are available in the supplementary material except for bird migration data, which are available at https://www.ebird.org.

## Supplementary material

For supplementary material accompanying this paper visit http://doi.org/10.1017/S0950268822001856. click here to view supplementary material
